# Effect of Boiling Time on the Color, Water, Protein Secondary Structure, and Volatile Compounds of Beef

**DOI:** 10.3390/foods14081372

**Published:** 2025-04-16

**Authors:** Liqin You, Yanfeng Zhang, Yingjuan Ma, Yongrui Wang, Zhaojun Wei

**Affiliations:** 1 College of Biological Science and Engineering, North Minzu University, Yinchuan 750021, China; youliqin2016@163.com (L.Y.);; 2 Specialty Food Nutrition and Health Innovation Team of Ningxia Hui Autonomous Region, North Minzu University, Yinchuan 750021, China; 3 School of Animal Science and Technology, Ningxia University, Yinchuan 750021, China

**Keywords:** beef, boiling process, GC–MS, water distribution, protein

## Abstract

The influence of boiling time on the persistent changes in the surface color, water content and distribution, protein secondary structure, and the concentration of volatile compounds in beef were studied, in order to obtain quality short-term boiled beef slices. The results show that the water content of beef samples significantly decreased and migration occurred between the high-freedom water and the low-freedom water. On average, boiling for 1 min was a key point in the changes of color parameters (L*, a*, b*, *w*, Δ*E,* and *BI*) and partial protein secondary structure because of the change in the ambient temperature around beef. In six samples, 29 volatile compounds were confirmed by GC–MS, and 13 compounds were regarded as the potential key volatile compounds, including 1-heptanol, 1-octen-3-ol, octanal, hexanal, decanal, heptanal, nonanal, (*E*, *E*)-2,4-decadienal, (*E*, *E*)-2,4-nonadienal, dodecanal, (*E*)-2-undecenal, 2,3-octanedione, and 2-pentylfuran. The color, water, and protein secondary structure were closely correlated with some potential key volatile compounds. The results could be used to guide the consumers to better grasp the quality of hot-pot meat during gatherings and have a comfortable consumer experience.

## 1. Introduction

Beef is highly praised and advocated for its high protein content, high-quality fatty acids, and distinct sensory experience [1]. Sensory aspects, such as odor, appearance, texture, and taste, can affect consumers’ overall like and buying desire for beef [2]. The content and composition of intramuscular fat (IMF) in beef are critical for the formation of beef flavor after hot processing [3]. Marble pattern is a white adipose tissue spot or stripe within the muscle fiber bundle of skeletal muscle, which reflects the external manifestation of intramuscular fat, and its formation is influenced by various factors. In beef cattle, IMF accumulation is greatly influenced by genetic background [4]. Japan Wagyu are recognized as the best quality beef breeds in the world today, with distinct marble patterns on their meat, also known as “snowflake meat”. Qinchuan cattle is the main species in some northwest provinces of China, such as Ningxia Hui Autonomous Region, Shanxi Province, and Gansu Province. In the recent years, the crossbred cattle between Japanese Wagyu and Qinchuan cattle of the second generation (CCWQ) were cultivated to improve the quality of local Chinese beef.

Meat flavor is a critical quality evaluation indicator for consumers before consumption [5]. Generally, raw meat exhibits a bloody and metallic aroma [6]. Many intricate reactions occur during meat cooking, thereby producing a huge number of volatile compounds. These compounds in meat are primarily derived from a series of complex chemical reactions during meat processing, including Maillard reaction, lipids oxidation, and the lipid–Maillard interaction. The desirable colors, mouthfeel, umami, and flavors of meat processing originate from these complex chemical reactions [7]. Lipid oxidation is the main source of flavor for cooked meat, producing over half of the flavor compounds [8]. The unsaturated fatty acids like oleic acid, linoleic acid, linolenic acid, and arachidonic acids are the most common fatty acids in livestock and poultry meat, and more than 50% of the volatile compounds in cooked meat are produced by the oxidization and degradation of these fatty acids [8]. Usually, octanal, nonanal, and 2-undecenal are mainly generated from the oxidation of oleic acid [9]; 2-nonenal, hexanal, (*E*, *E*)-2,4-decadienal, and 2-pentylfuran are mainly produced by the oxidation of linoleic acid [10]; and 1-octene-3-ol is mostly formed via the oxidation of arachidonic acid [11]. The Maillard reaction is a reaction that occurs between reducing sugars and amino compounds when reaching a certain temperature [12]. The products of the Maillard reaction frequently include oxygen-containing heterocyclic compounds, sulfur-containing compounds, and nitrogen-containing heterocyclic compounds [13]. Furthermore, the lipid–Maillard interaction also participates in the formation of volatile compounds of the cooked meat [14]. The aroma of meat products varies with different hot processing methods, and heating temperature and time play an important role in it [15].

Water is the main component of muscle tissue in raw meat, accounting for over 75% of the total mass [16]. The changes in hydrogen bonding between water molecules, ions, and biomacromolecules, like protein and lipids molecules, may result in change in the meat’s texture and flavor. Research has found that ketones, alcohols, aldehydes, and esters can bind to proteins via hydrophobic interactions. The change in water content in meat can alter this hydrophobic interaction, resulting in a change in volatile compounds [17]. In addition, the loss of water means a decrease in meat weight, resulting in economic losses [17]. Therefore, it is necessary to master the dynamic changes in water in meat during processing.

Proteins can contribute to flavor release because of their ability to combine with volatile compounds [18]. This ability on the binding of proteins and flavor compounds has been widely studied and numerous authors have even tried to to analyze it using model solutions. The ability of carnosine, anserine, and myoglobin to interact with some key flavor compounds like hexanal, 3-methyl-butanal, octanal, 2-methyl-butanal, methional, and 2-pentanone was investigated by Gianelli et al. (2003) [19]. Qi et al. (2018) found that thermally degraded gelatin increased surface hydrophobicity and flavor binding through hydrophobic interactions [20]. The binding ability between silver carp myosin and flavor compounds is positively correlated with ion strength, and exhibits the strongest binding ability at neutral pH [21]. On the basis of the primary structure, protein molecules are coiled and folded, and form protein secondary structures under the fixation of hydrogen bonds in the molecule [22]. Once the ability of proteins to bind to aromatic compounds changes, the perception of meat’s aroma can be deeply influenced [20]. The original conformation of peptides is disrupted during heating, leading to increased thermal motion, loss of secondary and tertiary structures, intermolecular force breakage, such as electrostatic or non-polar interactions, and disulfide bonds [23]. Therefore, the sensory quality of cooked meat, such as tenderness and aroma, is influenced by changes in protein denaturation and fiber structure caused by heat treatment [24]. Whether meat is suitable for cooking mainly depends on macro factors. However, the microstructural changes in meat after cooking are closely related to the macroscopic changes in meat, and so far, these changes have received little attention [3].

Hot-pot (huǒguō), one traditional Chinese dish that originated in Sichuan and Chongqing, is favored by consumers in China and around the world for its unique taste and flavor [25]. As an essential ingredient in hot-pot cooking, the beef is usually sliced thinly and is briefly boiled in boiling water before consumption. The common cooking method of beef in daily life is cooking in large chunks, which takes longer to cook. The beef in the form of thin slices makes it easier to cook. However, prolonged boiling can also lead to a deterioration in the taste of beef. At present, there is little research on the changes in quality during the boiling process of beef slices and beef rolls. Hence, this study aimed to (i) analyze the color change in beef during boiling process; (ii) examine volatile compounds of boiled beef during the processing through GC–MS combined with chemometric analysis and electronic nose analysis; (iii) investigate the key aroma compounds in boiled beef based on odor activity values (OAVs); (iv) study the water content and distribution of boiled beef during boiling process; and (vi) analyze the changes in the protein secondary structure of boiled beef during boiling process.

## 2. Materials and Methods

### 2.1. Reagents and Chemicals

Beef sample from the longissimus dorsi muscle of the CCWQ (twenty-four months old) was purchased from Yuxiang Co., Ltd. (Shizuishan, China). The following chemicals were purchased from Sigma-Aldrich (Shanghai, China): hexanal (95.0%), nonanal (99.5%), octanal (99.0%), 2-pentylfuran (98.0%), 1-octen-3-ol (98.0%), (*E*)-2-undecenal (96.0%), dodecanal (92.0%), 1-heptanol (97.0%), decanal (98.0%), heptanal (97.0%), 1,2-dichlorobenzene (internal standard, 99.78%), and n-alkanes (C_7_-C_40_, ≥97.0%). (*E*, *E*)-2,4-decadienal (94.0%) and (*E*, *E*)-2,4-nonadienal (95.8%) were from TCI Development Co., Ltd. (Shanghai, China); 2,3-octanedione (≥97.0%) was purchased from Macklin Biochemical Co., Ltd. (Shanghai, China). Methanol (analytical grade) was purchased from Thermo Fisher Scientific Co., Ltd. (Shanghai, China).

### 2.2. Raw Material and Sample Preparation

The beef blocks were cut into 3.0 × 3.0 × 0.1 cm pieces. The pieces were poured into a stainless steel slotted spoon and cooked in boiling water for 0, 1, 3, 5, 7.5, 10 min. Each sample was repeated three times.

### 2.3. Color Analysis

The CR-400 Chroma Meter (MINOLTA Co., Ltd., Osaka, Japan) was used to measure the color (L*, a*, b*) of boiled beef samples. They were placed in the room for 15 min to cool to room temperature (25 ± 1 °C) after boiling. The standard white plate was used to calibrate the instrument prior to measurement. The measurement of meat’s surface was taken in three color parameters L* (lightness), a* (red–green chromatic), and b* (yellow–blue chromatic). The whiteness (*w*), total color difference (Δ*E*), and browning index (*BI*) of all boiling beef samples were evaluated according to the formula described by Wang et al. [17].

### 2.4. Water Analysis

The total water content was determined by the direct drying method [26]. The water state and distribution in beef samples were analyzed on an NMI20-NMR analyzer (Niumag Co., Ltd., Shanghai, China). Three relaxation times (*T*_21_, *T*_22,_ and *T*_23_) as well as their respective relaxation signal components (*M*_21_, *M*_22,_ and *M*_23_) were recorded under the conditions of the Carr–Purcell–Meiboom–Gill (CPMG) sequence, and the parameters were the same as our earlier study [17].

### 2.5. Analysis of Volatile Compounds

#### 2.5.1. Electronic Nose Analysis

The PEN 3.5 E-nose (Airsense, Schwerin, Germany) was used to distinguish the volatile compounds in different boiling samples. The 5.00 g sample was accurately weighed in a 10 mL glass vials, and equilibrated at 50 °C water bath for 30 min prior to analysis. The electronic nose parameters were the same employed by Wang et al. [17].

#### 2.5.2. GC–MS Analysis

The GC–MS system (GC-MS 2010 plus, SHIMADZU, Kyoto, Japan) equipped with a DB-WAX capillary column (30 m × 0.25 mm × 0.25 µm, Agilent Technologies, Santa Clara, CA, USA), was used to analyze the aroma compounds. Before extracting the volatile compounds of beef samples, the 50/30 m DVB/CAR/PDMS SPME fiber was inserted into the 250 °C injection port for 90 min to ensure that there were no residues. The sample bottle containing 2 g of beef was put into a 55 °C water bath for volatile compound equilibrium, with an equilibrium time of 20 min. After equilibrium, the 50/30 m DVB/CAR/PDMS SPME fiber was inserted into the headspace of the sample bottle for 30 min. After 30 min of absorption, the SPME fiber was quickly transferred to the GC inlet for 5 min of desorption at 250 °C. The temperature programs of the oven in GC–MS were previously explained by Wang et al. [17].

#### 2.5.3. Quantitation and OAVs Analysis of Volatile Compounds

Qualitative analysis of flavor compounds in beef was conducted using three methods: the NIST 14 database (MS), retention indices with reference values (RI), and authentic volatile standards (S). The key volatile compounds were quantified by standards with 5-point curves, and other volatile compounds without authentic volatile standards were quantified by 1,2-dichlorobenzene (4 µL, 6.42 µg/mL). The final concentration of each flavor compound was divided by the reported odor threshold to obtain OAV. The contribution rate was the OAVs ratio of each flavor compound to all flavor compounds.

### 2.6. Analysis of Protein Secondary Structure

Boiled beef was vacuum-frozen in a FD-12A-110 vacuum freeze drier (Shunzhi Co., Ltd., Shanghai, China) under the conditions of −25 °C freezing temperature, −30 °C cold trap temperature, and 20 Pa vacuum, dried continuously for 48 h, and then crushed into 200 mesh powder. A Perkin Elmer Spectrum Two FTIR spectrometer (Perkin Elmer, CT, USA) was used to analyze the protein secondary structure of beef samples. The wave number range for scanning samples was 400–4000 cm^−1^ and the 1600–1700 cm^−1^ range was selected for protein secondary structure analysis. The peak of protein secondary structure corresponding to amide I was as follows: α-helix (1663–1646 cm^−1^), β-sheet (1700–1682 cm^−1^ and 1636–1615 cm^−1^), β-turn (1681–1664 cm^−1^), and random coil (1645–1637 cm^−1^) [27].

### 2.7. Statistical Analysis

The color data of beef samples were expressed as means ± standard deviation in the table after a variance analysis (ANOVA) using SPSS 19.0 software (IBM Corporation, New York, NY, USA). Differences among individual means were analyzed by Duncan’s multiple range test (*p* < 0.05). The PCA of electronic nose data was carried out by the software of SIMCA14.0. The protein secondary structure was analyzed using Omnic 8.2 and Peakfit 4.12 software (Systat Software Inc., San Jose, CA, USA). All graphs were made by the website (http://www.bioinformatics.com.cn/, accessed on 15 September 2024) and Origin 18C software.

## 3. Results

### 3.1. Changes in the Water Content of Boiled Beef

**Figure 1:**
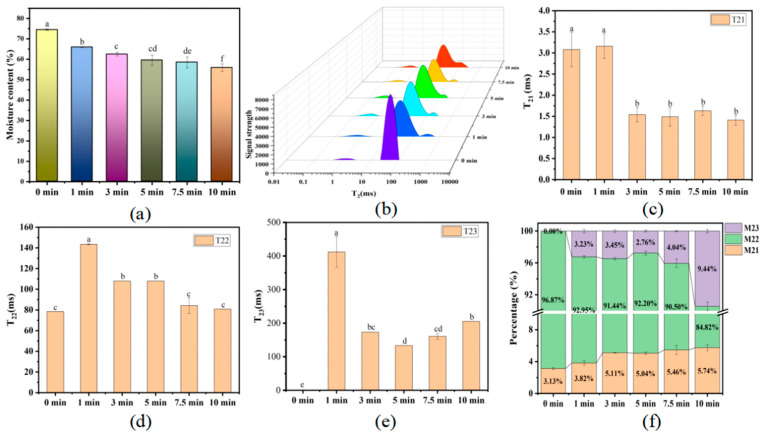
Change in the water content and water distribution of boiled beef under different boiling times. (**a**) Change in the water content of boiled beef during boiling. (**b**) *T*_2_ distribution curve of the boiled beef during boiling. (**c**) Change in the *T*_21_ value of boiled beef during boiling. (**d**) Change in the *T*_22_ value of boiled beef during boiling. (**e**) Change in the *T*_23_ value of boiled beef during boiling. (**f**) Change in percentage content of three different waters in boiled beef during boiling. Different letters in the same row indicates statistical difference (*p* < 0.05) with ANOVA using Duncan’s multiple range test.

Water content of boiled beef refers to the remaining water in beef after boiling, also reflecting the juiciness of beef. Generally, more than 70% of the weight in beef (raw) is water, and the largest proportion is immobilized water [28]. As shown in Figure 1a, the water content of beef gradually decreased with the boiling time increasing, with only 55.89% after 10 min of boiling.

LF-NMR technology was commonly applied in the food fields and other fields with fast, accurate, non-destructive, and non-invasive features, and can display the water state and distribution in samples. The state of water in boiled beef was described using relaxation times (*T*_21_, *T*_22,_ and *T*_23_) and corresponding percentages (*M*_21_, *M*_22,_ and *M*_23_) of peak areas (Figure 1b). Three peaks (*T*_21_, *T*_22_, and *T*_23_) were observed and referred to the proportion of three different water [17]. Peaks with *T*_21_ values between 0.01 and 10 ms are regarded as bound water, with *T_22_* values between 10 and 100 ms representing immobilized water, and with *T*_23_ values between 100 and 1000 ms defined as free water. The changes in *T*_21_, *T*_22,_ and *T*_23_ relaxation times of boiled beef with increasing boiling time are shown in Figure 1. In total, the peak positions of the three waters shifted from the high relaxation times to the low relaxation times, and the peak height of *T_22_* was significantly decreased during 0–1 boiling time. The value of *T*_21_ and *T*_22_ increased during 0–1 boiling time and decreased during 1–10 min boiling time. The values of *T*_23_ in the samples were significantly increased for 0–1 min, then decreased for 1–5 min, and finally significantly increased for 5–10 min. The peak areas of *M*_21_ and *M*_23_ increased and the peak area of *M*_22_ decreased with the increase in boiling time.

### 3.2. Changes in the Surface Color Value of Beef

The surface color of the cooked meat can intuitively reflect important information, such as the quality (flavor and texture) and safety (shelf life) [10]. Obviously, the color of the boiled beef changed from bright red to gray white. This appearance was obvious and could be directly observed by the naked eyes. As shown in Table 1, compared with raw meat, the L* and b* increased in the samples boiled for 1 min, and then remained stable in the samples boiled for 1–10 min. Meanwhile, the a* of samples showed a completely opposite trend. The *w*, Δ*E,* and *BI* were further obtained. The changes in *w* and Δ*E* were consistent with the L* and b*, while the changes in *BI* were consistent with a*.

### 3.3. Volatile Compounds

#### 3.3.1. E-Nose Analysis

**Figure 2:**
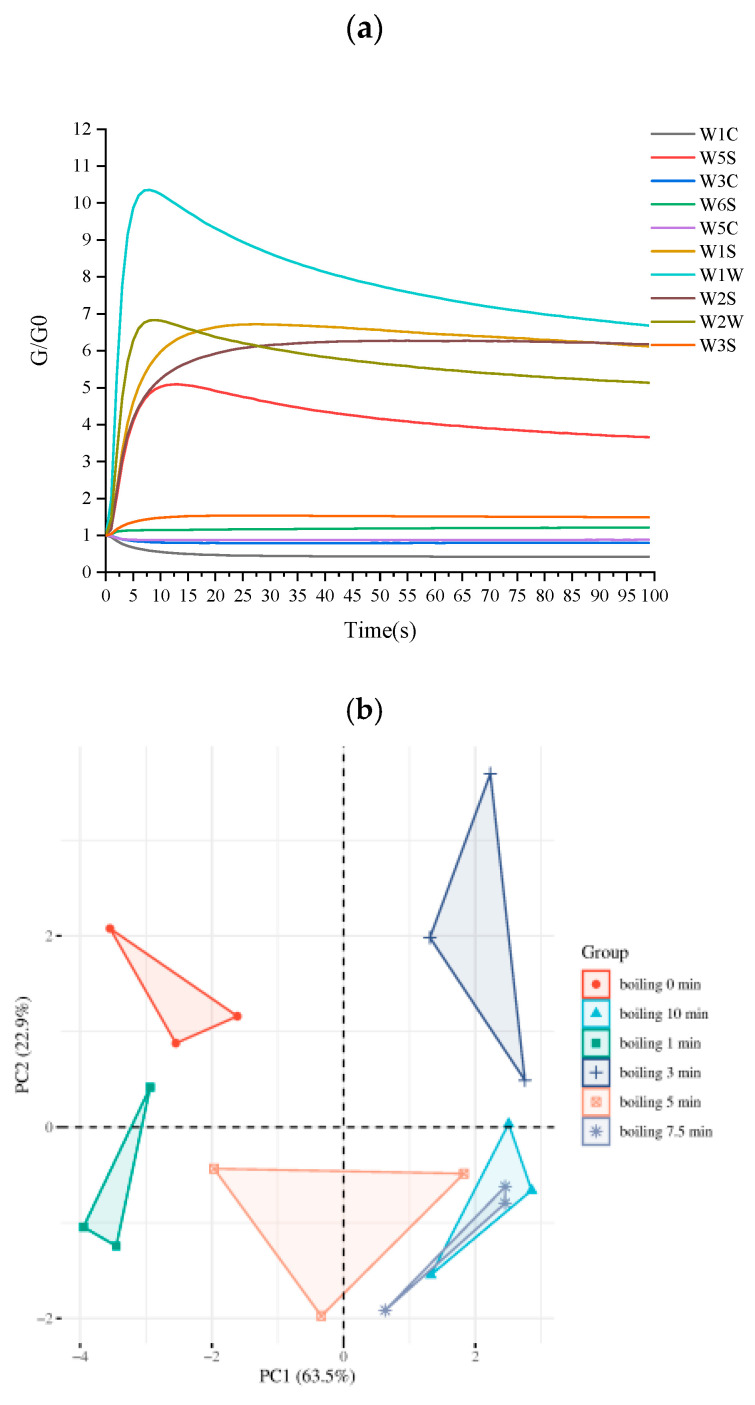
Analysis of E-nose data. (**a**) Radar plots of the responses of E-nose sensors to boiled beef. (**b**) Principal components analysis of E-nose data of boiled beef with different boiling time. The sensors included W1C (aromatic compounds), W3C (ammonia, aromatic compounds), W5S (nitrogen oxides), W6S (hydrocarbons), W5C (alkanes and aromatics), W1S (methane, broad range of compounds), W1W (sulfur compounds, terpenes), W2S (broad range, alcohols), W2W (aromatics and organic sulfur compounds), and W3S (methane and aliphatic compounds).

An E-nose combined with principal component analysis (PCA) was used to distinguish the six beef samples. The response of the E-nose to the samples of beef is shown in Figure 2a. The strong responses generated from the W1W, W2W, W5S, and W1S receptors indicate that the volatile compounds in boiled beef samples contain high concentrations of nitrogen and sulfides oxides. However, there were differences in the signal strength of various sensors in the E-nose between beef samples. PCA was used to analyze the E-nose data of boiled beef (Figure 2b). The first two principal components contributed as much as 86.4% (PC1 63.5%, PC2 22.9%) to the cumulative variance, meaning that the majority of information for all samples was covered. The differences between beef samples were mainly concentrated near PC1, and E-nose completely separated the six sample groups. However, this requires further analysis to determine the contribution of the compounds to the boiled beef samples.

#### 3.3.2. Volatile Compounds Identified by GC–MS

**Figure 3:**
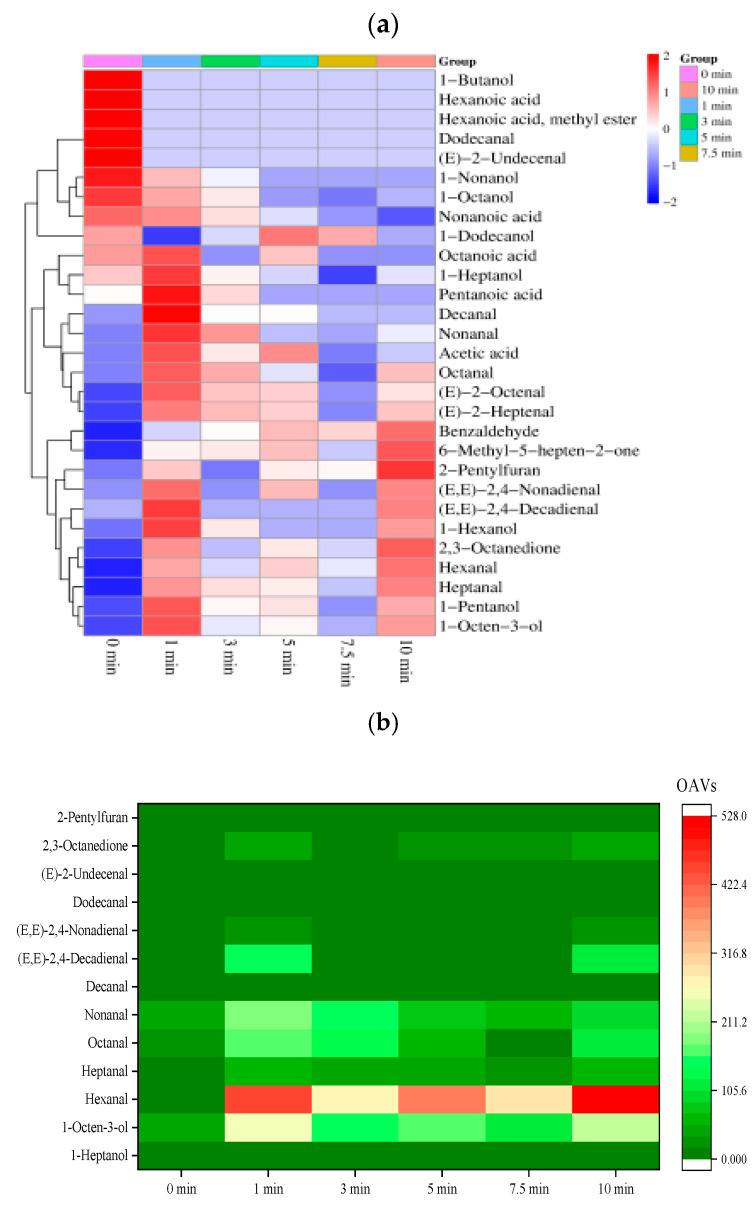
Analysis of volatile compounds in boiled beef. (**a**) Clustering heatmap of the contents of volatile compounds in boiled beef. (**b**) Changes in OAVs of aroma compounds (OAVs > 1) in boiled beef during the boiling process.

GC–MS was applied to analyze the volatile compounds of boiled beef. A total of 29 volatile compounds in six samples of boiled beef were identified, including 12 aldehyde, 8 alcohol, 2 ketone, 5 acid, 1 ester and 1 heterocyclic compounds (Figure 3a). The total content of all volatile compounds quantitated by standards ranged from 326.68 to 3796.11 μg/kg, and the total content in the raw beef sample was the lowest. With the increase in boiling time, the total content increased from 0 to 1 min of boiling, decreased from 1 to 7.5 min, and finally increased slightly at 10 min. Among these odorants, 24, 23, 20, 21, 18, and 20 odorants were identified in the beef at 0, 1, 3, 5, 7.5, and 10 min of boiling, respectively. In the raw meat, 1-butanol, hexanoic acid methyl ester, (*E*)-2-undecenal, and hexanoic acid were identified, whereas they disappeared after boiling for 1 min. Inversely, (*E*)-2-heptenal and benzaldehyde only appeared in the boiled samples. The concentrations of 1-octen-3-ol, 2,3-octanedione, nonanal, 1-pentanol, heptanal, 1-hexanol, hexanal, decanal, and 6-methyl-5-hepten-2-one in the beef boiled for 1–10 min were significantly higher (*p* < 0.05) than those in the raw meat.

In all samples, whether raw or boiled beef, aldehydes, alcohols, and ketones were the main compounds with higher variety and concentration. Hexanal, heptanal, nonanal, and octanal were the major aldehydes and widely present in various beef samples; 1-octen-3-ol was the pivotal alcohol and 2,3-octanedione was the pivotal ketone in boiled beef. Compared to the raw meat, most aldehydes, alcohols, and ketones significantly increased in boiled beef after boiling for 1–10 min. In particular, hexanal (2369.81 μg/kg), heptanal (173.45 μg/kg), and 2,3-octanedione (501.57 μg/kg) predominantly contributed to the aroma in the boiled beef after boiling for 10 min, while 1-pentanol (173.22 μg/kg), 1-hexanol (63.25 μg/kg), 1-octen-3-ol (257.02 μg/kg), octanal (119.69 μg/kg), nonanal (181.22 μg/kg), and acetic acid (48.56 μg/kg) predominantly contributed to the aroma in boiled beef after boiling for 1 min.

Not all volatile compounds exhibit a certain intensity of odor and make a significant contribution to the overall odor of meat, depending on their concentration and threshold. Generally, the compounds with OAVs > 1 can be considered as the key volatile compounds. As shown in Figure 3b, 13 of the volatile compounds identified had OAVs > 1, including 9 aldehydes, 2 alcohols, 1 ketone, and 1 furan (1-heptanol, 1-octen-3-ol, octanal, hexanal, decanal, heptanal, nonanal, (*E*, *E*)-2,4-decadienal, (*E*, *E*)-2,4-nonadienal, dodecanal, (*E*)-2-undecenal, 2,3-octanedione, and 2-pentylfuran). In addition, 10, 11, and 11 out of 13 compounds were discovered in the raw meat, in the beef boiled for 1 min and 10 min with OAVs greater than 1, respectively. It is especially important that a total of six compounds with OAV values greater than 100 were present, including octanal, 1-octen-3-ol, heptanal, nonanal, hexanal, and (*E*, *E*)-2,4-decadienal, indicating that these substances play a significant role in the flavor of boiled beef.

### 3.4. Change in Protein Secondary Structure in Boiled Beef

**Figure 4:**
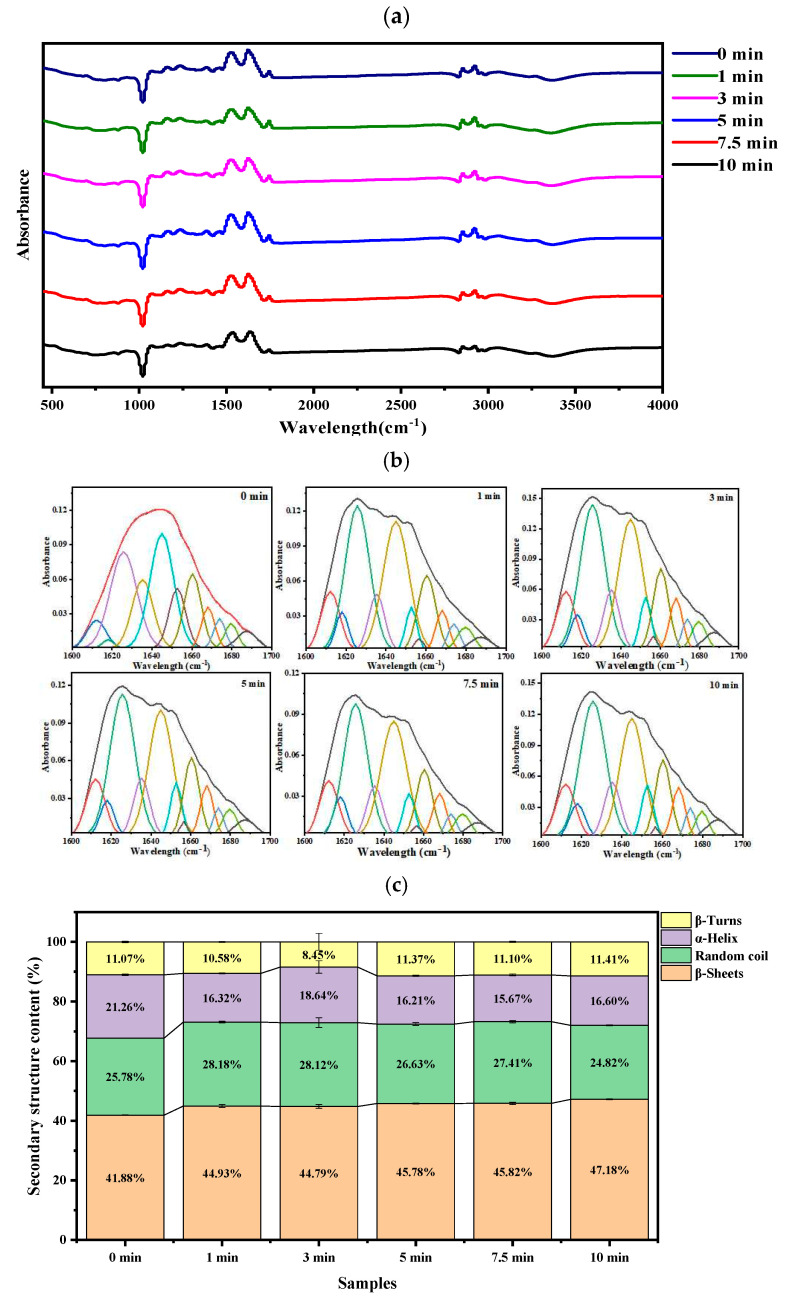
Infrared spectral analysis of the protein secondary structural composition of boiled beef. (**a**) Changes in FTIR spectroscopy (400–4000 cm^−1^) of boiled beef during boiling. (**b**) Decomposition of the boiled beef in the region of 1600–1700 cm^−1^. (**c**) Changes in the relative contents of protein secondary structures of beef (α-helix, β-sheets, β-turns, and random coil) during boiling.

FTIR spectroscopy was used to detect the changes in the secondary structure of boiled beef samples. As shown in Figure 4, the α-helix content significantly decreased (*p* < 0.05) and the β-sheet content significantly increased in the β-sheet (*p* < 0.05) during 0–1 min of boiling, and both remained stable after 1 min of boiling. However, the contents of random coils and β-turn in all the beef samples were not discernibly changed.

## 4. Discussion

### 4.1. The Changes in Water Content

Water plays an important role in both the metabolic activities of living animals and the post mortem processing of animals. For meat products during hot processing, water has a crucial impact on the texture (such as juiciness, chewiness), flavor, and color of the meat [29]. In this study, the water content decreased with the increase in time. This is because proteins undergo denaturation during thermal processing, causing transverse or longitudinal contraction of muscle fibers, resulting in the formation of gaps between muscle fibers. The binding between muscle fibers and water was weakened, making it easier for the water in the beef to be lost. In addition, this resulted in the loss of some soluble components, such as soluble protein, fat, and non-protein components. The decrease in water content further illustrates that water loss accounts for a greater proportion of the loss of meat quality [30]. The rise in *T*_21_, *T*_22,_ and *T*_23_ in the samples boiled for 0–1 min may be due to the gradual changes in beef when raw meat is rapidly exposed from room temperature to boiling water at 100 °C. The relaxation time of *T_2_*_1_ was observed to be decreased in the beef samples boiled for 1–10 min. A shorter relaxation time reflects lower water mobility, while a longer *T*_2_ value indicates that water mobility is difficult to restrict, leading to migration [31]. Cooked meat exhibited a faster relaxation behavior, and *T*_22_ relaxation time may indicate the degree of interaction between water molecules and highly organized protein structures [32]. The *T*_22_ value of meat after heat treatment decreased [32]. *T*_22_ decreased in the beef samples boiled for 1–10 min, consistent with the report of Wang et al. [17], suggesting that water restricted in the myofibrillar network of boiled beef may flow into the extra-myofibrillar network region due to myofibril contraction. The *M*_22_ of the boiled beef decreased as the boiling time increased, suggesting the structural alterations of myofibrils due to meat shrinkage and toughening, as well as water loss from the intermyofibrillar gaps during boiling [17]. The increase in cooking time, myofibrillar contraction, and protein denaturation may reduce binding to water molecules. The changes in *M*_22_ and *M*_23_ indicate that the immobilized water entrapped in the myofibrillar network was converted into free water located in the intercellular space. Therefore, the water migrated from immobilized water to free water [33]. The decrease in *M*_22_ during the boiling process may result in the increase in *M*_21_, because compared to bound water, immobilized water is more likely to be lost during the processing.

### 4.2. The Changes in Color

Color is an important physical indicator for consumers when purchasing food [33]. The changes in water content and optical transparency caused by protein thermal denaturation can affect the L* of meat [34]. In the beginning of the boiling process (0–1 min), the myosin is gradually destroyed and the ferrous ions in hemoglobin are oxidized, resulting in an increase in L* [35]. Meanwhile, sarcoplasmic and myofibrillar proteins under high temperature conditions undergo denaturation and aggregation, leading to increased light scattering [35]. The changes in a* during the early boiling process (0–1 min) were mainly due to the increased oxidative denaturation of myoglobin when the beef was in a high-temperature environment [36]. The formation of myoglobin may be the main reason for the increase in b* [35]. After 1 min of boiling, the protein of the beef surface completely denatures. At the boiling temperature, the caramelization reaction did not occur, so the color value of beef remained basically unchanged. This is also the reason why the *w*, Δ*E,* and *BI* did not change significantly after 1 min of boiling.

### 4.3. Key Aroma Compounds in Boiled Beef

Many studies have confirmed that alcohols and aldehydes are the main aroma compounds of muscle products [27,37,38,39,40]. Among the 29 components identified in all beef samples, there were 12 aldehydes and 8 alcohols, and 11 of them were regarded as key volatile compounds of the boiled beef. These alcohols and aldehydes are mainly saturated or unsaturated compounds, especially some C_6_–C_10_ compounds with high concentration and OAVs, including 1-heptanol, octanal, 1-octen-3-ol, nonanal, hexanal, heptanal, decanal, (*E*, *E*)-2,4-decadienal, and (*E*, *E*)-2,4-nonadienal. In total, alcohols and aldehydes contributed 95.96–98.88% of OAVs to the key aroma compounds of boiled beef in the process of boiling for 0–10 min; 1-octen-3-ol showed the highest OAVs (48.93) and highest contribution rate (34.41%) in raw meat, followed by octanal and nonanal. Unlike raw meat, hexanal had the highest contribution rate in all the boiled samples, reaching its peak in 7.5 min of boiling (55.66%) and the contribution rate of 1-octen-3-ol ranks second. This was consistent with a previous report showing that some volatile compounds, like octanal, hexanal, heptanal, and 1-octen-3-ol, generated from lipid oxidation had the higher concentrations in samples of home cooking processes [41]. The formation of aldehydes and alcohols was closely related to fatty acids, mainly unsaturated fatty acids in meat [37]. It was reported that octanal, 1-heptanol, decanal, (*E*, *E*)-2,4-nonadienal, hexanal, heptanal, nonanal, (*E*)-2-undecenal, (*E*, *E*)-2,4-decadienal, dodecanal, and 1-octen-3-ol were formed from the breakdown of linoleic acid and α-linolenic acid [27]. Furthermore, 2,3-octanedione and 2-pentylfuran that might also be formed from the decomposition of lipid were also observed in the boiled beef samples with OAVs > 1 [42].

The boiled beef exhibited the typical meaty and fatty aromas mainly caused by the 13 key volatile compounds. Hexanal, with the highest concentration in the boiled beef samples, showed a fatty aroma [38]. In addition, the ratio of hexanal to nonanal was proposed as an indicator of mutton freshness and overall quality [26]. The octanal was responsible for the meaty, grassy, and fresh aromas [37]. The decanal exhibited fatty, rancid, meaty, and burnt aromas, and the dodecanal presented lily, fatty, and citrus aromas [8]. Other key linear aldehydes, including nonanal and heptanal, mainly produced a grassy aroma [38]. As for unsaturated aldehydes, (*E*)-2-undecenal showed orange and green aroma, (*E*, *E*)-2,4-decadienal presented plastic and tailing odor, and (*E*, *E*)-2,4-nonadienal presented fatty and bad odor [8]. The 1-octen-3-ol was the degradation product of the secondary hydroperoxides of fatty acids and displayed a strong mushroom flavor [43], 1-heptanol presented floral aromas and was the key volatile compound in roasted chicken [27], and 2-pentylfuran presented a floral–fruity aroma and may significantly contribute to meat aroma [44]. The concentrations of 13 key volatile compounds increased significantly after the boiling, which produced abundant aromas in the beef samples. This result is consistent with the report by Cheng et al. (2023), who found that the formation of the volatile compounds in meat came from the thermal processing process [45]. The synergism of 13 key volatile compounds dominated the total aroma of the boiled beef, and other compounds (OAVs < 1) may have a modifying effect on the overall aroma of the boiled beef samples.

### 4.4. Protein Secondary Structure in Boiled Beef

Like the color value, the secondary structure of proteins in beef samples does not change significantly, and the peak patterns of the IR spectrum at 1600–1700 cm^−1^ are similar after 1 min of boiling. A high energy input (heating with high temperature) may result in a significant destruction and denaturation in the protein secondary structure. The presence of β-sheet secondary structure makes protein structures more stable and more fibrous [46,47]. As shown in Figure 3b, the peak near 1650 cm^−1^ was considered as the α-helix in the infrared spectrum of the beef sample, indicating a high proportion of α-helix in raw meat sample. The peak in the infrared spectrum shifted from the α-helix region to the β-sheet region (near 1624 cm^−1^) during the boiling process of beef, indicating that the β-sheet content had gradually increased. The β-sheet content significantly increased, and the α-helix content significantly decreased with the extension of boiling time, consistent with the findings of previous studies [48,49]. An increase in the β-sheet content and a decrease in the α-helix content represented protein unfolding and aggregation, respectively [50]. The heating process can enhance the kinetic energy level of protein to increase its “thermal motion”, causing the intermolecular forces (such as disulfide bonds, nonpolar interactions, and various kinds of electrostatic interactions) to be disrupted, which leads to the destruction of the native conformation (denaturing) of a protein [51]. As the heating time increases, the protein begins to unfold, and when the secondary structure is almost lost, they may aggregate due to side-chain modification.

### 4.5. Relationship Between Color, Water, Protein Secondary Structure, and Key Aroma Compounds

**Figure 5:**
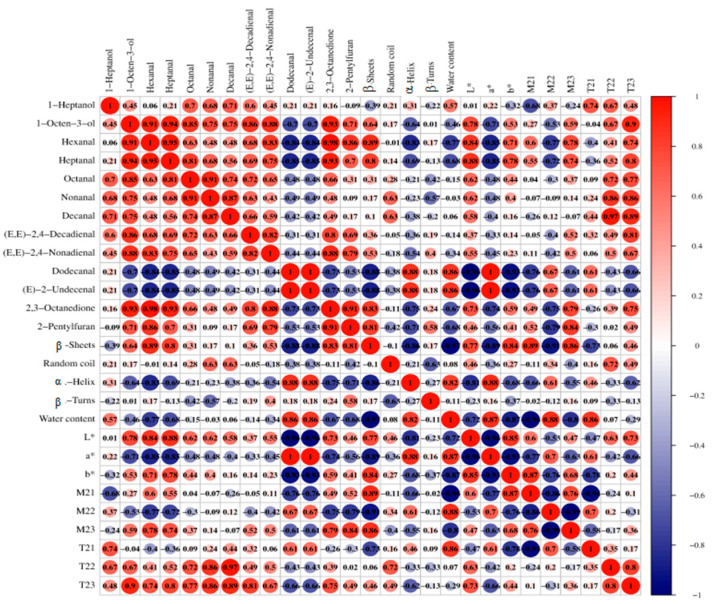
Correlation analysis among water content, color, protein secondary structure content, and levels of key volatile compounds. The red color represents positive correlation, and the blue color represents negative correlation.

Many flavor compounds were verified as binding to proteins via hydrophobic interactions, and this binding ability can be influenced by protein conformation (α-helix, β-sheet, and β-turn) [39]. Furthermore, some flavor compounds generated from the Maillard reaction are related to the surface temperature of meat during cooking and greatly affect color [35]. As shown in Figure 5, β-sheet was negatively correlated with the level of dodecanal and (*E*)-2-undecenal, and positively correlated with the content of hexanal, heptanal, 2,3-octanedione, and 2-pentylfuran. At the same time, α-helix content was positively correlated with the content of dodecanal and (*E*)-2-undecenal, and negatively correlated with the level of hexanal. During the boiling process, the contents of β-sheets, hexanal, heptanal, 2,3-octanedione, and 2-pentylfuran tended to rise, while the contents of α-helix and (*E*)-2-undecenal tended to decrease. This is mainly attributed to the denaturation of proteins and oxidation of fats [45]. The changes in color in 0–1 min of the boiling process may be attributed to the denaturation of proteins. The L* was negatively correlated with the level of dodecanal and (*E*)-2-undecenal, and positively correlated with the contents of hexanal and heptanal. Conversely, the a* had a r positive correlation with the level of dodecanal and (*E*)-2-undecenal, and showed a negative correlation with the content of hexanal and heptanal. The b* was negatively correlated with the dodecanal and (*E*)-2-undecenal level. In addition, water distribution and mobility have a significant impact on meat quality characteristics, affecting the content and composition of volatile flavor compounds in meat products as well [40]. *T*_23_ shows a positive correlation with the level of 1-octen-3-ol, heptanal, nonanal, and decanal, *T*_22_ has a positive correlation with the content of nonanal, decanal, and (*E*, *E*)-2,4-decadienal, and *M*_23_ shows a positive correlation with the level of 2-pentylfuran. In summary, during the boiling process, the color, flavor, water distribution, and protein secondary structure of the beef tended to be correlated.

## 5. Conclusions

The law of the persistent changes in the surface color, water content and distribution, protein secondary structure, and the changes in volatile compounds in the boiled beef were investigated in this work. The water content of the beef samples significantly decreased during the boiling process. The parameters of color and protein secondary structure of the beef remained almost unchanged after 1 min of boiling, indicating that the protein in the beef has been entirely denatured. Based on the GC–MS and OAVs analysis, 13 key volatile compounds were identified, including 1-heptanol, 1-octen-3-ol, hexanal, nonanal, heptanal, octanal, decanal, (*E*, *E*)-2,4-decadienal, (*E*, *E*)-2,4-nonadienal, dodecanal, (*E*)-2-undecenal, 2,3-octanedione, and 2-pentylfuran. During the boiling process, the changes in flavor, protein, color, and water content may affect the taste and smell of the beef. When beef is boiled for too short a time, the meat may have a bloody smell and there are also factors that can lead to diseases, such as liver hydatid disease. When beef is boiled for too long, the meat may harden and affect the taste. Therefore, an appropriate boiling time can result in a better sense of smell and taste of beef. Therefore, the results of this study may provide a theoretical basis for the appropriate boiling time of beef slices in hot-pot. We will further perform in-depth research on the key volatile compounds of traditional Chinese condiments or hot-pot seasoning to establish a complete system for revealing the contribution of condiments to the aroma of boiled beef and condiments in the hot-pot, which is helpful to develop the best taste and flavor for hot-pot.

## Figures and Tables

**Table 1:** Change in the color of boiled beef under different boiling times.

Boling Time (min)	0	1	3	5	7.5	10
L*	37.89 ± 1.66 ^d^	57.90 ± 2.08 ^a^	56.10 ± 1.09 ^ab^	57.48 ± 1.33 ^a^	52.46 ± 2.49 ^c^	54.09 ± 1.49 ^bc^
a*	27.44 ± 2.51 ^a^	11.40 ± 0.46 ^b^	11.55 ± 0.35 ^b^	11.45 ± 0.15 ^b^	11.29 ± 0.69 ^b^	10.95 ± 0.23 ^b^
b*	6.33 ± 1.66 ^d^	9.35 ± 0.31 ^c^	11.27 ± 0.57 ^bc^	9.96 ± 0.30 ^bc^	10.15 ± 0.51 ^abc^	10.77 ± 0.65 ^ab^
*w*	31.80	55.39	53.23	54.85	50.09	51.59
Δ*E*	0	25.82	24.67	25.55	22.08	23.54
*BI*	46.32	14.05	15.06	14.33	15.60	14.82

Results are expressed as the mean ± standard deviation. The *w*, Δ*E*, and *BI* represent whiteness, total color difference, and browning index, respectively. Different letters in the same row indicates statistical difference (*p* < 0.05) with ANOVA using Duncan’s multiple range test.

## Data Availability

The original contributions presented in this study are included in the article. Further inquiries can be directed to the corresponding authors.
